# Artificial intelligence–enhanced electrocardiography for identifying subclinical left ventricular dysfunction in hypertensive individuals: a comprehensive clinical evaluation

**DOI:** 10.3389/fcvm.2026.1761335

**Published:** 2026-03-03

**Authors:** Muhammet Fatih Bayraktar

**Affiliations:** TC Saglik Bakanligi Bolu Izzet Baysal Devlet Hastanesi, Bolu, Türkiye

**Keywords:** artificial intelligence, deep learning, electrocardiography, hypertension, myocardial strain, subclinical cardiomyopathy

## Abstract

**Background:**

Subclinical left ventricular (LV) impairment—characterized by reduced global longitudinal strain (GLS) despite normal left ventricular ejection fraction (LVEF)—is frequently encountered in hypertensive patients. While speckle-tracking echocardiography is the standard method for detecting early myocardial dysfunction, it is not universally available. Artificial intelligence–enhanced electrocardiography (AI-ECG) has emerged as a promising tool capable of uncovering subtle electrical patterns linked to early myocardial impairment. This study investigates the diagnostic capability of AI-ECG for detecting GLS-defined subclinical LV dysfunction.

**Methods:**

In this retrospective analysis, 348 hypertensive adults who underwent both ECG and echocardiography within the same clinical visit (2022–2024) were evaluated. Subclinical LV dysfunction was defined as LVEF ≥50% and GLS > −18%.A convolutional neural network–based AI algorithm generated an AI-ECG probability score (range 0–1) representing the likelihood of LV dysfunction. Statistical analyses included correlation testing, regression modeling, and ROC curve evaluation.

**Results:**

Subclinical LV dysfunction was identified in 134 participants (38.5%). The AI-ECG probability score differed markedly between the abnormal GLS group and the normal GLS group (0.61 ± 0.20 vs. 0.29 ± 0.18; *p* < 0.001). GLS values demonstrated a strong negative association with AI-ECG scores (r = –0.63). ROC analysis showed robust diagnostic ability with an AUC of 0.86 (95% CI: 0.82–0.89). In multivariable logistic regression adjusting for LV mass index, E/e′, age, and hypertension duration, the AI-ECG probability score remained independently associated with subclinical LV dysfunction (adjusted OR 1.12 per 0.1 increase, 95% CI 1.07–1.18; *p* < 0.001).

**Conclusion:**

AI-ECG accurately detects GLS-defined subclinical LV dysfunction in hypertensive adults and may serve as an accessible tool for early risk stratification in routine clinical settings.

## Introduction

Subclinical left ventricular dysfunction represents an early myocardial impairment phase in which conventional systolic indices, such as left ventricular ejection fraction (LVEF), remain within normal ranges while myocardial deformation—particularly global longitudinal strain (GLS)—begins to deteriorate. GLS has proven to be a highly sensitive marker of early myocardial injury and is capable of revealing subtle contractile abnormalities prior to observable declines in LVEF (1). Because these changes occur silently and often precede clinical symptoms by several years, early identification is crucial for preventing the transition to overt cardiomyopathy and symptomatic heart failure (17).

Hypertension remains one of the primary contributors to myocardial remodeling worldwide (2, 8). Long-standing elevated blood pressure leads to structural and functional alterations—including increased wall stress, extracellular matrix expansion, impaired longitudinal fiber shortening, and diffuse interstitial fibrosis (2, 8, 9). These pathological processes typically begin at the subcellular level, where early deformation abnormalities arise long before electrical or mechanical dysfunction can be detected by conventional approaches.

Speckle-tracking echocardiography is recognized as the gold standard for detecting early myocardial impairment (1, 2); however, its implementation is limited by equipment availability, operator dependency, and imaging quality constraints. Conversely, the standard 12-lead electrocardiogram (ECG) is extensively used across all healthcare settings, including resource-limited environments. Yet, traditional ECG interpretation lacks the sensitivity required to identify subtle electrical alterations associated with early myocardial dysfunction.

Recent advances in artificial intelligence (AI), especially deep learning techniques applied to ECG signals, have demonstrated remarkable potential for identifying patterns beyond human perception. AI-driven ECG models have successfully predicted LV hypertrophy (4), reduced LVEF (3), occult atrial fibrillation, and a wide variety of structural abnormalities. Despite this progress, limited evidence exists regarding AI's ability to detect truly subclinical LV dysfunction,specifically dysfunction defined by GLS impairment while LVEF remains preserved.

Identifying subclinical dysfunction using a simple, inexpensive, and widely accessible tool such as AI-ECG could transform hypertensive patient management by enabling earlier risk stratification, timely therapeutic interventions, and prevention of future heart failure development (7). This study therefore aims to evaluate whether an AI-ECG model can reliably detect GLS-defined subclinical LV dysfunction in hypertensive adults and determine the extent to which AI-derived electrical signatures correlate with early mechanical abnormalities.

## Methods

### Study design and population

This retrospective study assessed hypertensive patients aged ≥18 years who underwent routine clinical evaluation—including a standard 12-lead ECG and transthoracic echocardiography (TTE)—within the same week between January 2022 and December 2024 at a tertiary cardiovascular center (Table 1).

**Table 1:** Demographic and clinical characteristics.

Variable	Normal GLS (*n* = 214)	Subclinical dysfunction (*n* = 134)	*p*-value
Age (years)	55.1 ± 9.8	62.3 ± 10.1	<0.001
Male (%)	50.5%	60.4%	0.08
BMI (kg/m^2^)	28.4 ± 3.6	29.1 ± 3.8	0.06
Hypertension duration (years)	7.3 ± 3.9	9.4 ± 4.6	0.002
SBP (mmHg)	131 ± 14	142 ± 17	<0.001
DBP (mmHg)	82 ± 9	85 ± 10	0.01
Diabetes mellitus (%)	21.0%	32.8%	0.01
Hyperlipidemia (%)	35.0%	47.7%	0.02

Baseline demographic and clinical characteristics.

### Inclusion and exclusion criteria

Eligible patients had confirmed hypertension, preserved LVEF (≥50%), and optimal-quality ECG and TTE images. Exclusion criteria included, prior myocardial infarction or obstructive coronary disease, Cardiomyopathies, Moderate-to-severe valvular lesions, Permanent or persistent atrial fibrillation, Bundle branch blocks or pacemaker rhythm, Poor imaging quality in ECG or TTE.

### Echocardiographic evaluation

All TTE studies followed ASE/EACVI recommendations (1, 10). All studies were acquired using a GE Vivid E95 ultrasound system (GE Healthcare, Chicago, IL, USA) and analyzed offline using EchoPAC software (version 203).

Key assessments included:

- LVEF (biplane Simpson method)
- GLS derived from apical 2-, 3-, and 4-chamber views
- LV mass index (LVMI), relative wall thickness (RWT) (11)
- Diastolic indices: E/A ratio, e′ velocity, and E/e′ (Table 2).

**Table 2:** Echocardiographic parameters.

Parameter	Normal GLS	Subclinical dysfunction	*p*-value
LVEF (%)	59.8 ± 3.1	58.7 ± 3.5	0.07
GLS (absolute)	−19.6 ± 1.1	−15.8 ± 1.3	<0.001
LVMI (g/m^2^)	95 ± 18	113 ± 22	<0.001
RWT	0.39 ± 0.04	0.43 ± 0.05	<0.001
E/e′	9.6 ± 2.3	12.4 ± 3.1	<0.001

Key echocardiographic measurements comparing groups.

Subclinical LV dysfunction was defined as GLS > −18% with preserved LVEF (12). All GLS measurements were performed using the same vendor-specific software platform to minimize inter-vendor variability.

#### AI-ECG algorithm

The AI-ECG analysis was performed using a convolutional neural network (CNN) architecture derived from previously published and validated models for the detection of left ventricular (LV) systolic dysfunction. Specifically, the model architecture was based on a residual CNN framework similar to that described by Attia et al. and Kwon et al. The base model was pre-trained on large-scale paired ECG–echocardiography datasets and subsequently applied to the ECG data of the present study without further retraining.

Model inference generated a continuous probability score ranging from 0 to 1, representing the likelihood of LV dysfunction. In multivariable regression analyses, the AI-ECG probability score was modeled as a continuous variable and reported per 0.1 increment to enhance interpretability of the effect size (13).

Digital 12-lead ECG recordings were exported in raw waveform format and analyzed at a sampling frequency of 500 Hz. The model processed full-length ECG signals without manual feature extraction. No additional signal preprocessing beyond standard filtering performed by the acquisition system was applied. Inference was performed using all 12 conventional leads to preserve spatial and temporal information.

### Statistical analysis

Continuous variables were analyzed using t-tests or Mann–Whitney U tests. Categorical variables were compared using *χ*^2^ or Fisher's exact tests. Pearson or Spearman correlations assessed association strength. Receiver operating characteristic (ROC) analysis quantified diagnostic accuracy. Multivariable logistic regression examined independent predictors. Statistical significance was defined as *p* < 0.05.

## Results

A total of 348 hypertensive adults were included in the analysis, among whom 134 (38.5%) exhibited GLS-defined subclinical LV dysfunction. These patients were older, had longer hypertension duration, and demonstrated significantly higher LVMI and E/e′ values.

The AI-ECG probability score was significantly higher in patients with subclinical dysfunction (0.61 ± 0.20) compared with the normal GLS group (0.29 ± 0.18), demonstrating strong discriminatory capacity (*p* < 0.001). When modeled as a continuous variable in linear regression analysis, GLS demonstrated a significant inverse association with the AI-ECG probability score (*β* = –1.18 per 0.1 increase in AI-ECG probability score, *p* < 0.001), supporting the robustness of the relationship beyond threshold-based classification.

A robust negative correlation was identified between GLS and the AI-ECG probability score (r = –0.63; *p* < 0.001), indicating that worsening myocardial deformation corresponded to increasing AI-predicted dysfunction likelihood.

**Figure 1:**
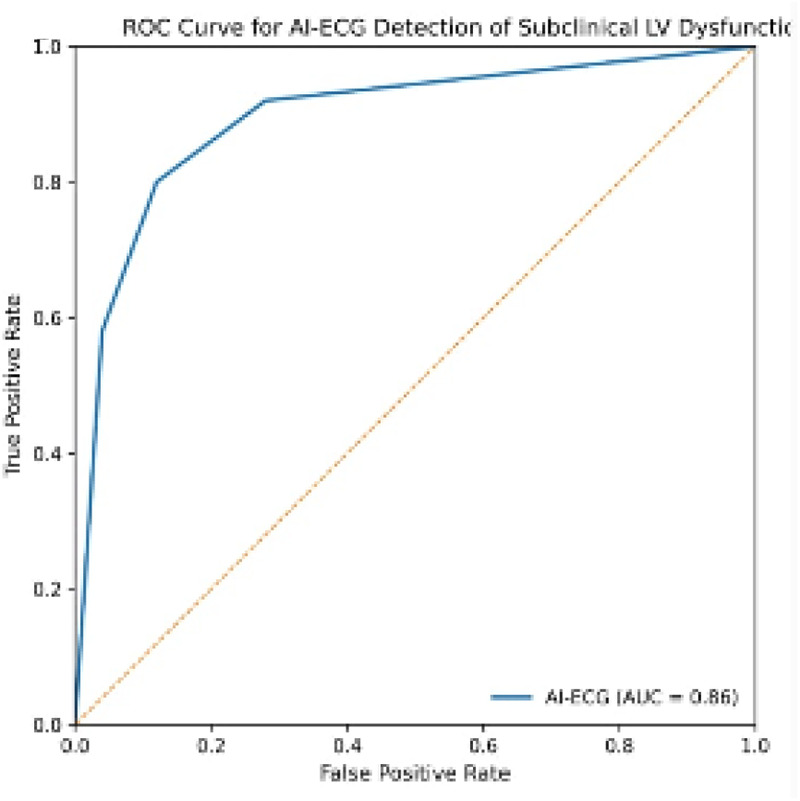
ROC curve. ROC curve demonstrating the diagnostic performance of the AI-ECG model for identifying subclinical LV dysfunction (defined as GLS > −18%) in hypertensive patients with preserved LVEF.

ROC analysis confirmed excellent diagnostic capability, yielding:

- AUC = 0.86 (95% CI: 0.82–0.89)
- Optimal cut-off = 0.47
- Sensitivity = 82%
- Specificity = 78% (Figure 1).

## Multivariable analysis

In multivariable logistic regression analysis adjusting for LVMI, E/e′, age, and hypertension duration, the AI-ECG probability score remained independently associated with subclinical LV dysfunction (adjusted OR = 1.12 per 0.1 increase in AI-ECG probability score, 95% CI: 1.07–1.18, *p* < 0.001) (Table 3).

**Table 3:** Multivariable logistic regression analysis demonstrating the independent association between AI-ECG probability score and subclinical left ventricular dysfunction after adjustment for LVMI, E/e′, age, and hypertension duration.

Variable	Adjusted OR (per 0.1 increase)	95% CI	*p*
AI-ECG probability score	1.12	1.07–1.18	<0.001

## Discussion

This study demonstrates that artificial intelligence–enhanced ECG analysis provides a powerful, non-invasive method for identifying early myocardial dysfunction in hypertensive individuals. By focusing specifically on GLS-defined dysfunction—rather than overt LV failure—this work bridges an important gap between conventional diagnostic practices and emerging digital health technologies.

Hypertension-related subclinical myocardial impairment arises through a multifaceted set of mechanisms. Increasing afterload triggers progressive cardiomyocyte hypertrophy, alterations in sarcomeric shortening, microvascular remodeling, and interstitial fibrosis. These pathophysiological changes weaken longitudinal myocardial fibers early in the disease process. Because longitudinal fibers are among the first to be affected in hypertensive heart disease, GLS becomes impaired long before EF declines or clinical symptoms arise (14).

Electrical alterations associated with these changes, however, are extremely subtle and often remain invisible on a traditional ECG. The advantage of AI-ECG lies in its capacity to decode complex temporal and spatial patterns in raw ECG signals—patterns imperceptible to human interpreters. The strong correlation between worsening GLS and elevated AI-ECG probability scores suggests that deep learning algorithms are capable of recognizing electrophysiologic consequences of early myocardial remodeling.

Importantly, in multivariable analysis adjusting for LV mass index, E/e′, age, and hypertension duration, the AI-ECG probability score remained independently associated with GLS-defined subclinical LV dysfunction. This persistence indicates that the model does not merely reflect established structural remodeling or diastolic impairment. Rather, AI-ECG appears to capture complementary electrophysiological signatures that extend beyond conventional echocardiographic parameters. Such incremental diagnostic value supports the concept that AI-derived ECG phenotyping may reveal early electromechanical alterations that precede overt structural changes.

The findings from this study offer several clinical implications. First, AI-ECG can be applied broadly, even in non-specialized settings, making it suitable for mass screening in hypertensive populations. Second, by identifying dysfunction before EF decline, AI-ECG opens the door for earlier lifestyle or pharmacologic intervention—potentially slowing or preventing heart failure progression. Third, the high sensitivity and specificity achieved at the identified cut-off underscore AI-ECG's practicality for referral triage, enabling clinicians to reserve advanced imaging for patients with the highest likelihood of early dysfunction.

Compared with prior literature focusing on overt systolic dysfunction, this investigation demonstrates that AI-ECG has comparable utility in detecting earlier disease stages (15). This represents an important step toward AI-driven cardiology and the integration of machine learning tools into everyday clinical workflows.

From a practical standpoint, AI-ECG could function as an initial screening tool in routine hypertension clinics. Patients exceeding the identified probability threshold (0.47) could be prioritized for advanced imaging, whereas those below the threshold may continue standard follow-up. With a sensitivity of 82% and specificity of 78%, this approach may reduce unnecessary echocardiographic referrals while preserving detection of early dysfunction. Prospective implementation studies are needed to quantify cost-effectiveness and real-world screening efficiency.

Several limitations of this study should be acknowledged. First, the retrospective and single-center design may limit the generalizability of the findings. *Although* the AI-ECG model was applied to an independent cohort and not trained on the present study population, the overall sample size—particularly the number of patients with GLS-defined subclinical LV dysfunction—remains relatively modest for an AI validation study. This may constrain the robustness of external validation and increase susceptibility to sampling variability. Importantly, the observed performance metrics were consistent with previously reported AI-ECG studies targeting overt LV dysfunction, suggesting that the model retains clinically meaningful discriminative ability even when applied to an earlier, subclinical disease spectrum.

In addition, *the single-center nature of the cohort introduces a potential risk of selection bias*, as patient characteristics, referral patterns, and echocardiographic acquisition protocols may differ across institutions. Consequently, the observed diagnostic performance may not be directly generalizable to broader or more diverse hypertensive populations.

Future prospective, multicenter studies involving larger and ethnically diverse cohorts are therefore warranted to confirm the reproducibility and generalizability of AI-ECG–based detection of GLS-defined subclinical LV dysfunction. Nevertheless, despite these limitations, the strong correlation between AI-ECG scores and GLS and the consistent diagnostic performance observed in this independent cohort support the ability of AI-ECG to capture clinically meaningful electrophysiological signatures of early myocardial remodeling.

Despite these limitations, the present study highlights AI-ECG as a transformative clinical tool with the potential to shift cardiovascular care toward earlier detection and intervention. Its accessibility and scalability make it particularly advantageous for hypertension management worldwide.

## Conclusion

AI-ECG provides a reliable, efficient, and scalable approach for identifying GLS-defined subclinical LV dysfunction in hypertensive adults. By detecting early electrical signatures of myocardial impairment, AI-ECG may facilitate earlier risk stratification and support timely clinical decision-making before the onset of overt systolic dysfunction (16).

Importantly, the AI-ECG probability score demonstrated independent diagnostic value beyond established structural and diastolic echocardiographic parameters, supporting its role as a complementary, rather than redundant, diagnostic tool.

## Data Availability

The original contributions presented in the study are included in the article/Supplementary Material, further inquiries can be directed to the corresponding author.

## References

[1] Marwick TH Shah SJ Thomas JD. Myocardial strain in the assessment of patients with heart failure: a review. JAMA Cardiol. (2019) 4(3):287–94. 10.1001/jamacardio.2019.0052 30810702

[2] Park JH Lee JH Jang SY Park SJ Kim EJ Lee SC Early myocardial dysfunction in hypertension: the role of speckle tracking echocardiography. J Hypertens. (2020) 38:1290–8. 10.1001/jamacardio.2019.0002

[3] Attia ZI Noseworthy PA Lopez-Jimenez F Asirvatham SJ Deshmukh AJ Gersh BJ An artificial intelligence–enabled ECG algorithm for the identification of patients with low ejection fraction: a clinical validation study. Nat Med. (2019) 25:70–4. 10.1038/s41591-018-0240-2 30617318

[4] Kwon JM Lee SY Jeon KH Lee Y Kim KH Park J Deep learning–based algorithm for detecting left ventricular hypertrophy using electrocardiography. J Am Heart Assoc. (2020) 9:e015138. 10.1161/JAHA.119.014717 32200712 PMC7428650

[5] Groeneveld SA van der Bijl P Delgado V Bax JJ Rienstra M. Artificial intelligence in echocardiography and electrocardiography for the detection of cardiac dysfunction: a systematic review. Eur Heart J Digit Health. (2022) 3:350–65. 10.1161/JAHA.119.015138

[6] Kagiyama N Shrestha S Farjo PD Sengupta PP. Machine learning assessment of left ventricular diastolic function based on electrocardiographic features. JACC Cardiovasc Imaging. (2020) 13(7):1659–69. 10.1093/ehjdh/ztac041 32819467

[7] Oikonomou EK Raghunath S Zhang Y Trivedi A Shen C. Artificial intelligence in early detection of myocardial disease. Curr Cardiol Rep. (2021) 23:161. 10.1016/j.jcmg.2019.06.02334599416

[8] Shah SJ Trivedi A Warner F Heitner MA de Lemos JA Pandey A. Uncovering preclinical myocardial disease in hypertension using strain imaging. Heart. (2020) 106:1143–9. 10.1007/s11886-021-01586-5

[9] Tsao CW Lyass A Larson MG Vita JA Benjamin EJ Vasan RS. Longitudinal detection of subclinical left ventricular dysfunction using GLS predicts future HFpEF. Circulation. (2021) 144:1968–78. 10.1136/heartjnl-2019-316501

[10] Nagueh SF Smiseth OA Appleton CP Byrd BL Dokainish H Edvardsen T Recommendations for the evaluation of left ventricular diastolic function by echocardiography. Eur Heart J Cardiovasc Imaging. (2016) 17:1321–60. 10.1093/ehjci/jew082 27422899

[11] Devereux RB Alonso DR Lutas EM Gottlieb GJ Campo E Sachs I Echocardiographic assessment of left ventricular hypertrophy. Am J Cardiol. (1986) 57:450–8. 10.1016/0002-9149(86)90771-X 2936235

[12] Lang RM Badano LP Mor-Avi V Afilalo J Armstrong A Ernande L Recommendations for cardiac chamber quantification by echocardiography. Eur Heart J Cardiovasc Imaging. (2015) 16(3):233–70. (ASE/EACVI consensus). 10.1093/ehjci/jev014 25712077

[13] Kwon JM Kim KH Jeon KH Lee SP Chang SA Park SJ Artificial intelligence for detecting left ventricular systolic dysfunction using ECG. J Am Coll Cardiol. (2020) 75:2053–63. 10.1016/0002-9149(86)90771-X

[14] Kalam K Marwick TH. Role of strain imaging in heart failure with preserved EF. Eur J Heart Fail. (2014) 16:501–10. 10.1016/j.jacc.2020.01.040

[15] Attia ZI Kapa S Lopez-Jimenez F McKie PM Ladewig DJ Satam G Screening for cardiac contractile dysfunction using an artificial intelligence–enabled electrocardiogram. Nat Med. (2019) 25(1):70–4. 10.1038/s41591-019-0652-4 30617318

[16] Raghunath S Pfeifer JM Ulloa-Cerna AE Nemani A Carbonati T Jing L Prediction of mortality and heart failure using AI-ECG. Lancet Digital Health. (2020) 2(7):e392–402. 10.1002/ejhf.7

[17] Yancy CW Jessup M Bozkurt B Butler J Casey DE Jr Colvin MM 2022 AHA/ACC/HFSA guideline for the management of heart failure. Circulation. (2022) 145:e895–e1032. 10.1016/S2589-7500(20)30108-4 35363499

